# Proton-mediated photoprotection mechanism in photosystem II

**DOI:** 10.3389/fpls.2022.934736

**Published:** 2022-09-07

**Authors:** Yu Sugo, Hiroshi Ishikita

**Affiliations:** ^1^Department of Applied Chemistry, The University of Tokyo, Tokyo, Japan; ^2^Research Center for Advanced Science and Technology, The University of Tokyo, Tokyo, Japan

**Keywords:** photoprotection, photoinhibition, low-barrier hydrogen bond (LBHB), bicarbonate, proton-coupled electron transfer (PCET), formate, D1-Tyr246, Q_B_H_2_ release/exchange

## Abstract

Photo-induced charge separation, which is terminated by electron transfer from the primary quinone Q_A_ to the secondary quinone Q_B_, provides the driving force for O_2_ evolution in photosystem II (PSII). However, the backward charge recombination using the same electron-transfer pathway leads to the triplet chlorophyll formation, generating harmful singlet-oxygen species. Here, we investigated the molecular mechanism of proton-mediated Q_A_^⋅–^ stabilization. Quantum mechanical/molecular mechanical (QM/MM) calculations show that in response to the loss of the bicarbonate ligand, a low-barrier H-bond forms between D2-His214 and Q_A_^⋅–^. The migration of the proton from D2-His214 toward Q_A_^⋅–^ stabilizes Q_A_^⋅–^. The release of the bicarbonate ligand from the binding Fe^2+^ site is an energetically uphill process, whereas the bidentate-to-monodentate reorientation is almost isoenergetic. These suggest that the bicarbonate protonation and decomposition may be a basis of the mechanism of photoprotection *via* Q_A_^⋅–^/Q_A_H^⋅^ stabilization, increasing the Q_A_ redox potential and activating a charge-recombination pathway that does not generate the harmful singlet oxygen.

## Introduction

**FIGURE 1:**
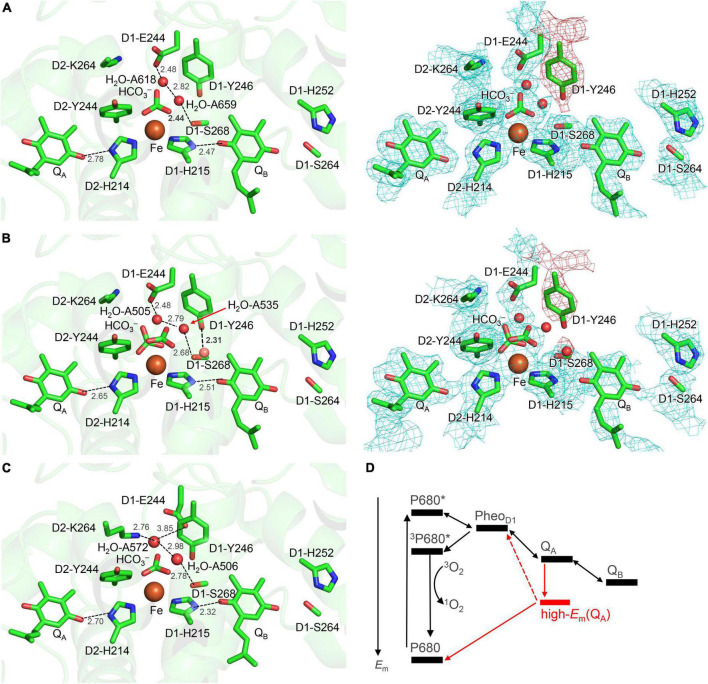
Overview of the quinone binding sites. **(A)** X-ray crystal structure of PSII from *Thermosynechococcus vulcanus* (PDB code, 3WU2) (Umena et al., 2011) and electron density map. **(B)** X-ray crystal structure of the PsbM-deleted PSII from *Thermosynechococcus vestitus* BP-1 (PDB code, 5H2F) (Uto et al., 2017) and electron density map. **(C)** Cryo-electron microscopy structure of PSII from *Synechocystis* sp. PCC 6803 (PDB code, 7N8O) (Ferreira et al., 2004). Dotted lines indicate H-bonds. The density of the D1-Tyr246 moiety, including the adjacent water molecule in the PsbM-deleted structure, is indicated by the red mesh. **(D)** Energetics of forward and backward electron-transfer processes. Processes occurring *via* the high-*E*_m_ Q_A_ form are shown in red. P680* denotes the electronically excited P680.

The driving force for photosynthetic O_2_ evolution is provided by the photo-induced charge separation at the reaction center of photosystem II (PSII). The electronic excitation of the chlorophyll leads to electron transfer *via* pheophytin in the D1 protein, Pheo_D1_, to two plastoquinone molecules, Q_A_ and Q_B_ (Shen, 2015). The terminal electron acceptor Q_B_ accepts two electrons *via* the primary quinone Q_A_ and two protons. The non-heme Fe^2+^ complex, which comprises D1-His215, D2-His214, D1-His272, D2-His268, and bicarbonate (HCO_3_^–^), is equidistant from Q_A_ and Q_B_ (Shevela et al., 2012; Figure 1A). The bidentate bicarbonate is modeled in the majority of the reported PSII structures (e.g., Umena et al., 2011; Gisriel et al., 2022; Figure 1A), whereas not only the bidentate but also monodentate bicarbonate is modeled in the PsbM-deleted PSII

(Figure 1B; Uto et al., 2017). D1-Tyr246 and D2-Tyr244 are located at the bicarbonate binding site (Forsman et al., 2019). The characteristic of D1-Tyr246 is the electron density near the hydroxyl group (Saito et al., 2013), which is clearly observed in the 1.9-Å X-ray diffraction (XRD) (Umena et al., 2011) and X-ray free electron laser (XFEL)-S_1_ (Suga et al., 2015) structures (Figure 1A). The experimentally observed electron density was not interpreted in 2011 and 2015 (Figure 1A). In 2017, Uto et al. (2017) modeled the corresponding density as a water molecule (*B*-factor: 40 Å^2^) forming a significantly short H-bond with the hydroxyl group of D1-Tyr246 (2.31 Å) (Figure 1B). Remarkably, the O_D1–Tyr246_…O_water_ distance of 2.3 Å is closer to the O…O distance for a typical Zundel (H_2_O…H^+^…H_2_O) cation (≈2.3 Å). The absence of the corresponding density at the D2-Tyr244 moiety may indicate a functional asymmetry between the Q_A_ and Q_B_ sides (Saito et al., 2013).

The redox potential (*E*_m_) values of –100 to –140 mV for one-electron reduction of Q_A_ (Q_A_/Q_A_^⋅–^) (Krieger and Weis, 1992; Johnson et al., 1995; Krieger et al., 1995; Ishikita and Knapp, 2005a; Shibamoto et al., 2009) and 90 mV for Q_B_/Q_B_^⋅–^ (Kato et al., 2016; De Causmaecker et al., 2019) indicate that exergonic electron transfer occurs under normal functional conditions. Reduced Q_B_ accepts the first proton *via* D1-Ser264 and D1-His252 at the distal carbonyl O site with respect to the non-heme Fe^2+^ complex (Ishikita and Knapp, 2005a; Saito et al., 2013; Ashizawa and Noguchi, 2014) and the second proton *via* D1-His215 at the proximal carbonyl O site (Saito et al., 2013, 2020; Kimura et al., 2020); thus, reduced Q_B_ forms Q_B_H_2_ and moves from PSII toward the quinone pool. The depletion of the bicarbonate from the non-heme Fe^2+^ reduces the rate of the electron transfer from Q_A_ to Q_B_ (Jursinic et al., 1976; Eaton-Rye and Govindjee, 1988) or the exchange of Q_B_H_2_ (Siggel et al., 1977; Sedoud et al., 2011).

Under strong light, the plastoquinone pool is fully reduced, and the Q_B_ binding site is unoccupied, which inhibits electron transfer and causes Q_A_^⋅–^ to accumulate in PSII (photoinhibition) (Vass et al., 1992; Noguchi, 2002). If the *E*_m_ gap between Q_A_ and Pheo_D1_ is small, backward electron transfer occurs in the form of charge recombination from Q_A_^⋅–^ *via* Pheo_D1_ to the cationic chlorophyll in the reaction center, forming triplet chlorophyll and generating harmful singlet-oxygen species (Rutherford and Krieger-Liszkay, 2001; Rutherford et al., 2012). Q_A_ has been reported to exhibit two forms, a low- and a high-*E*_m_(Q_A_) conformation (Figure 1D; Krieger and Weis, 1992; Johnson et al., 1995; Krieger et al., 1995). Q_A_ exists in the low-potential form under normal functional conditions. The high-*E*_m_(Q_A_) conformation can increase the *E*_m_ gap between Q_A_ and Pheo_D1_, preventing charge recombination *via* Pheo_D1_ and singlet-oxygen generation under strong light [photoprotection (Rutherford and Krieger-Liszkay, 2001; Rutherford et al., 2012)].

The molecular origin of the high-*E*_m_(Q_A_) form had long remained unsolved. Vass et al. (1992) proposed that the Q_A_^⋅–^ stabilization is mediated by protonation. In the 3.5-Å crystal structure of PSII (Ferreira et al., 2004), the OH group of D2-Thr217 forms an H-bond with Q_A_^⋅–^ but not with unprotonated neutral Q_A_ (Ishikita and Knapp, 2005a). Thus, H-bond donation of D2-Thr217 to Q_A_^⋅–^ could be a possible mechanism for the formation of the high-*E*_m_ conformation. However, the corresponding H-bond is unlikely to form in the crystal structure of PSII from *Thermosynechococcus vulcanus* (Umena et al., 2011) and the recent cryo-electron microscopy structure of PSII from *Synechocystis* sp. PCC 6803 (Gisriel et al., 2022). The aforementioned crystal structure shows that Q_A_ has D2-His214 at the proximal carbonyl O site and the backbone amide group of D2-Phe261 at the distal carbonyl O site as H-bond partners.

The non-heme Fe^2+^ complex might be involved in the photoprotection mechanism (Diner and Petrouleas, 1987; Muh et al., 2012). Notably, Brinkert et al. (2016) reported that the loss of the bicarbonate ligand from the non-heme Fe^2+^ complex leads to an increase of 75 mV in *E*_m_(Q_A_) in the presence of Q_B_; this suggests that the loss of the bicarbonate ligand is responsible for the formation of the high-*E*_m_(Q_A_) conformation. Recent studies have shown that photo-induced CO_2_ conversion is likely to occur from the bicarbonate ligand at Fe^2+^ (Shevela et al., 2020). Bicarbonate loss and photo-induced CO_2_ conversion likely constitute the basis of the photoprotection mechanism. Although the loss of the negatively charged bicarbonate (HCO_3_^–^) certainly increases *E*_m_(Q_A_), the distance between the bicarbonate ligand and Q_A_ is more than 6 Å (Umena et al., 2011). Moreover, whether the proton-mediated Q_A_^⋅–^ stabilization mechanism (Vass et al., 1992) is still relevant to the Q_A_^⋅–^ stabilization remains unclear.

To understand the mechanism of how the loss of the bicarbonate increases *E*_m_(Q_A_), we investigated the bicarbonate and Q_A_ binding sites in PSII using a quantum mechanical/molecular mechanical (QM/MM) approach based on the PSII crystal structure (Umena et al., 2011).

## Materials and methods

### Coordinates and atomic partial charges

The atomic coordinates were obtained from the X-ray crystal structure of PSII (PDB code, 3WU2) (Umena et al., 2011). The heavy-atom positions were fixed while the H-atom positions were optimized with CHARMM (Brooks et al., 1983). All titratable groups were ionized if not otherwise specified. D1-His337 was considered to be protonated (Nakamura and Noguchi, 2017). Atomic partial charges were obtained from the CHARMM22 (MacKerell et al., 1998) parameter set for amino acids and previous studies for cofactors (Saito et al., 2015), respectively.

### Quantum mechanical/molecular mechanical calculations

The unrestricted density functional theory method was employed with the B3LYP functional and LACVP* basis sets (LANL2DZ (double ζ quality basis set with the Los Alamos effective core potential) for Mn and Ca atoms and 6-31G* for other atoms) (Hay and Wadt, 1985) using the QSite (QSite, 2012) program. Counter ions were added to neutralize the system. In the QM region, all atomic coordinates were relaxed. In the MM region, the H-atom positions were energetically optimized, and the heavy-atom positions were fixed using the OPLS2005 force field because the MM region is used mainly to reproduce (long-distance) electrostatic interactions with the QM region and the heavy-atom positions in the MM region should remain unchanged with respect to those in the original crystal structure. The initial-guess wave functions were obtained using the ligand field theory (Vacek et al., 1999) implemented in the QSite program. Three QM regions were used: (i) [Q_A_, Q_*B*,_ the non-heme Fe complex (bicarbonate if applicable, Fe, and side chains of D1-His215, D1-His272, D2-His214, and D2-His268), side chains of D1-His252, D1-Ser264, and D2-Phe261 (including backbone)] for the analysis of the H-bond between Q_A_ and D2-His214; (ii) [Q_A_, Q_*B*,_ the non-heme Fe complex (bicarbonate, Fe, and side chains of D1-His215, D1-His272, D2-His214, and D2-His268), side chains of D1-Tyr246, D1-Ser264, and D1-His252, and the modeled water molecule adjacent to D1-Tyr246] for the analysis of the proton transfer toward the bicarbonate ligand; and (iii) [Q_A_, Q_*B*,_ the non-heme Fe complex (bicarbonate, Fe, and side chains of D1-His215, D1-His272, D2-His214, and D2-His268), side chains of D1-Tyr246, D1-Ser264, D1-His252, and protonated D1-Glu244, and water molecules in the H-bond network (water molecules A618 and A659 and the modeled water molecule adjacent to D1-Tyr246] for the analysis of the proton transfer from D1-Ser268 to the bicarbonate ligand.

All other protein units and cofactors were approximated by the MM force field (i.e., electrostatic influences are sufficiently considered in the MM region). Note that the residues in the proton transfer pathways must be included in the QM region to consider the formation/breakage of the covalent (H-)bonds during proton transfer. See Supplementary material for the atomic coordinates of the resulting QM region. As in a previous study (Chernev et al., 2011), the non-heme Fe complex was in a high-spin state of Fe^2+^, and the spin multiplicity of the system was set to *S* = 2 in calculations for neutral Q_A_ and *S* = 5/2 for Q_A_^⋅–^.

The QM/MM-optimized geometry was used as the initial geometry to analyze the potential energy profiles. For the analysis of the N_D2–His214_…H^+^…O_QA_ H-bond, the focusing H atom was moved along the N…O H-bond from 0.02 to 0.10 Å (e.g., 0.02 Å around the local energy minimum in some cases), after which the geometry was optimized by constraining the N…H^+^ and H^+^…O distances and energy was calculated. For the analysis of the bidentate-to-monodentate reorientation of the bicarbonate ligand, the focusing O_HCO3–_…Fe distance was increased by 0.10 Å, after which the geometry was optimized by constraining the O_HCO3–_…Fe distance and energy was calculated. For the analysis of the release of the bicarbonate ligand from the Fe site, the C_HCO3–_…Fe distance was increased by 0.10 Å, after which the geometry was optimized by constraining the C_HCO3–_…Fe distance and energy was calculated. For the analysis of the proton transfer from H_3_O^+^ *via* D1-Tyr246 and the bicarbonate ligand, the focusing H atom was moved away from the electron donor O site from 0.02 to 0.10 Å, after which the geometry was optimized by constraining the O_donor_…H distance and energy was calculated. For the analysis of the proton transfer from H_3_O^+^ adjacent to D1-Ser268 to the bicarbonate ligand, the focusing H atom was moved to the electron acceptor O site by 0.10 Å, after which the geometry was optimized by constraining the O_acceptor_…H distance and energy was calculated. For the analysis of the protonated bicarbonate decomposition to H_2_O + CO_2_, the H_2_O…CO_2_ distance was increased by 0.10 Å, after which the geometry was optimized by constraining the H_2_O…CO_2_ distance and the energy was calculated.

## Results

### Formation of a low-barrier H-bond between D2-His214 and Q_A_ upon bicarbonate loss

**FIGURE 2:**
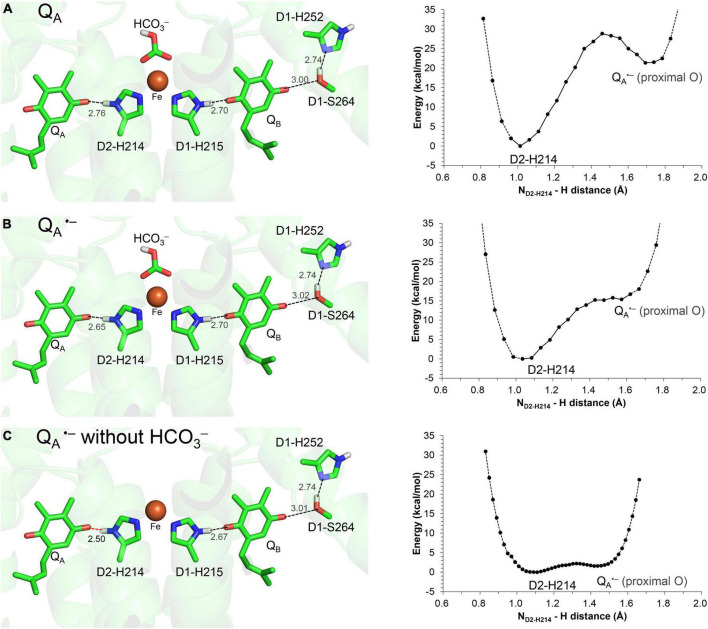
QM/MM-optimized geometry and potential energy profile for the H-bond between Q_A_ and D2-His214 prior to electron transfer from Q_A_ to Q_B_. **(A)** Neutral Q_A_. **(B)** Q_A_^⋅–^. **(C)** Q_A_^⋅–^ in the absence of the bicarbonate ligand. D1-Ser264 forms an H-bond with unprotonated D1-His252, as the electron is not transferred to Q_B_ (Ishikita and Knapp, 2005a). Note that the geometry of each intermediate conformation is fully QM/MM optimized.

The H-bond distance of 2.78 Å between D2-His214 and Q_A_ in the PSII crystal structure (Umena et al., 2011; Figure 1A) is closest to 2.76 Å for neutral unprotonated Q_A_ in the QM/MM-optimized geometry (Figure 2A). This suggests that Q_A_ is neutral Q_A_ in the PSII crystal structure. Although the H-bond is shortened to 2.65 Å when Q_A_ is reduced to Q_A_^⋅–^, the potential-energy profile indicates that the H^+^ is localized at the D2-His214 moiety (Figure 2B).

Remarkably, the D2-His214…Q_A_^⋅–^ H-bond transforms into a low-barrier H-bond (2.50 Å) in response to the loss of the bicarbonate ligand (Figure 2C). In addition to the electrostatic contribution of the loss of the negative charge to an increase in *E*_m_(Q_A_), the migration of the D2-His214 proton toward Q_A_^⋅–^ along the low-barrier H-bond (0.23 Å) stabilizes Q_A_^⋅–^ significantly. In addition to the loss of a negative charge, this is likely a substantial reason for the 75 mV upshift in *E*_m_(Q_A_) upon the loss of the bicarbonate (Brinkert et al., 2016) because Q_A_…HCO_3_^–^ is not short (6.8 Å).

The formation of the low-barrier H-bond between Q_A_^⋅–^ and D2-His214 suggests that p*K*_a_(Q_A_^⋅–^/Q_A_H) ≈ p*K*_a_(D2-His214-NH/N^–^) in the absence of the bicarbonate. The low-barrier H-bond formation is also observed between D1-His215 and Q_B_H^–^ in PSII (Saito et al., 2013) [and His-L190 and Q_B_H^–^ in photosynthetic reaction centers from purple bacteria (PbRC) (Sugo et al., 2021)] during the Q_B_H_2_ formation; this suggests that p*K*_a_(Q_B_H^–^/Q_B_H_2_) ≈ p*K*_a_(D1-His215-NH/N^–^) in the presence of the bicarbonate. p*K*_a_(D1-His215-NH/N^–^) and p*K*_a_(D2-His214-NH/N^–^) for deprotonation of singly protonated to anionic histidine are likely higher [e.g., 13 in the Rieske cluster (Zu et al., 2003; Hsueh et al., 2010)] than p*K*_a_ for deprotonation of doubly protonated to singly protonated histidine [e.g., 2–9 in protein environments (Grimsley et al., 2009)]. As p*K*_a_(QH^–^/QH_2_) = 11 for plastoquinone (Q) in water (Hasegawa et al., 2017), a slight decrease in p*K*_a_(D2-His214-NH/N^–^) due to Fe^2+^ can lead to p*K*_a_(Q_a_^⋅–^/Q_A_H) ≈ p*K*_a_(D2-His214-NH/N^–^).

In the low-barrier H-bond, the proton can move easily between the two H-bond moieties (Ishikita and Saito, 2014). In the low-barrier H-bond between TyrZ and D1-His190, the 0.35 Å migration of the proton toward TyrZ from D1-His190 can increase *E*_m_(TyrZ) by 130 mV (Saito et al., 2020). From the analogy, the > 0.2 Å migration of the proton toward Q_A_^⋅–^ along the low-barrier H-bond (Figure 2C) is the most likely origin of the observed increase of 75 mV in *E*_m_(Q_A_) (Brinkert et al., 2016). Thus, Q_A_^⋅–^ can be stabilized irrespective of Q_A_…HCO_3_^–^ = 6.8 Å.

### Energetics of the bicarbonate displacement from the non-heme Fe complex

**FIGURE 3:**
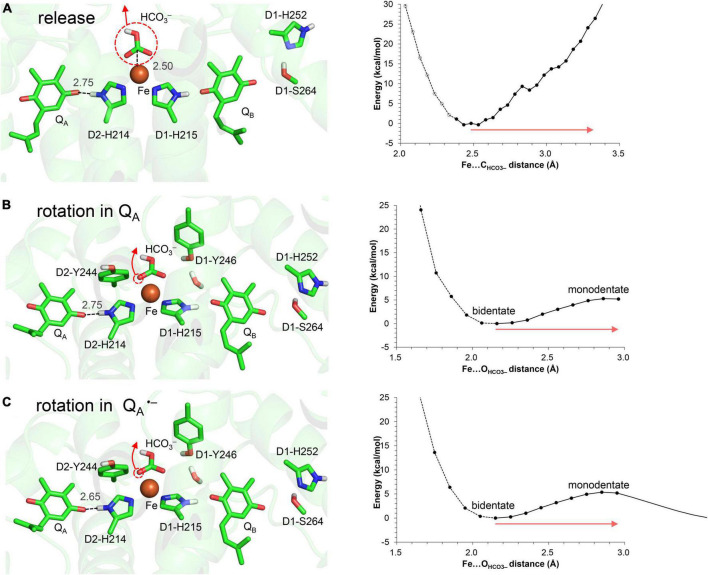
Energy profile for the bicarbonate displacement. **(A)** Release of the bicarbonate ligand from the Fe^2+^ site. **(B)** Bidentate-to-monodentate reorientation of the bicarbonate ligand in neutral Q_A_. **(C)** Bidentate-to-monodentate reorientation of the bicarbonate ligand in Q_A_^⋅–^. Red arrows indicate the orientations of the bicarbonate displacement. Note that the geometry of each intermediate conformation is fully QM/MM optimized.

In QM/MM calculations, the release of the bicarbonate ligand from the binding Fe^2+^ site is an energetically uphill process (Figure 3A), which suggests that the electrostatic interaction between Fe^2+^ and HCO_3_^–^ is not negligibly small as long as the negatively charged HCO_3_^–^ state is stable at Fe^2+^.

In contrast, the bidentate-to-monodentate reorientation is slightly uphill in Q_A_/Q_A_^⋅–^, as the monodentate bicarbonate can accept an H-bond from D1-Tyr246 (Figures 3B,C). Note that Q_A_^⋅–^ is stabilized when the hydroxyl group of D1-Tyr246 is oriented toward Q_A_^⋅–^ (Saito et al., 2013). It has been proposed that bicarbonate serves as not only a bidentate but also a monodentate ligand (Hienerwadel and Berthomieu, 1995; Chernev et al., 2011; Uto et al., 2017). In vacuum, the monodentate ligation may become pronounced specifically upon the formation of Q_A_^⋅–^ (Chernev et al., 2011), because the reorientation of the free bicarbonate ligand occurs easily and is the only way to compensate for the electrostatic repulsion against Q_A_^⋅–^. The corresponding electrostatic repulsion is smaller (bicarbonate…Q_A_^⋅–^ = 6.8 Å) than the interactions with D1-Glu244 (3.4 Å from bicarbonate), D1-Tyr246 (3.2 Å), and D2-Tyr244 (3.1 Å) in the PSII protein environment. The shape of the potential-energy profile remains essentially unaffected in response to changes in the Q_A_ redox state (Figures 3B,C). Furthermore, the bidentate ligation is more stable than the monodentate ligation in the PSII protein environment (Figures 3B,C). Thus, the bidentate-to-monodentate reorientation is unlikely to synchronize with unimpaired electron transfer from Q_A_ to Q_B_: the reorientation may occur only when the electron transfer is sufficiently slow to compete with the energetically uphill movement of the bicarbonate ligand.

These results suggest that the release of the bicarbonate ligand is energetically more uphill than the reorientation, as long as HCO_3_^–^ is ligated to the Fe^2+^ site.

## Discussion

### Possible mechanisms of bicarbonate loss

The bicarbonate protonation and decomposition can also lead to the bicarbonate loss (e.g., Loerting et al., 2000). This requires the proton transfer pathway toward the bicarbonate binding site. Below we have discussed candidate proton-donor residues near the bicarbonate binding site.

**FIGURE 4:**
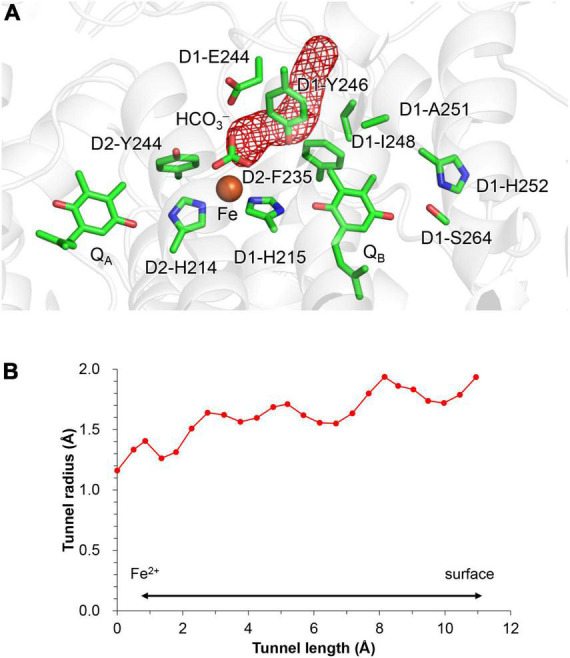
Channel that proceeds from the bicarbonate binding site toward the stromal protein surface. **(A)** Overview of the hydrophobic channel (red mesh). **(B)** Cavity radius along the hydrophobic channel. The channel space was analyzed using CAVER (Chovancova et al., 2012).

Notably, the bicarbonate binding site is linked with the protein bulk surface *via* an H-bond network that is formed by D1-Glu244, D1-Tyr246, and D1-Ser264 (Figure 4). D1-Glu244 in the highly charged *de*-loop region was responsible for the pH dependence of *E*_m_ for the non-heme Fe complex (Ishikita and Knapp, 2005b) and may be involved in the bicarbonate protonation at lower pH in the Fe^2+^ state (Kato et al., 2021). In addition, the *E*_m_(Q_A_) shift was observed upon the D1-E244A mutation (Forsman et al., 2019). Indeed, D1-Glu244 is close to the bicarbonate (3.37 Å, Figure 1) (Umena et al., 2011). However, according to the geometry of the PSII crystal structure, ionized D1-Glu244 is stabilized by a salt bridge with D2-Lys264 (3.31 Å, Umena et al., 2011) and is unlikely to serve as a proton donor to the bicarbonate. Unless structural changes occur at higher pH, the geometry does not directly support the involvement of D1-Glu244 in the bicarbonate protonation.

**FIGURE 5:**
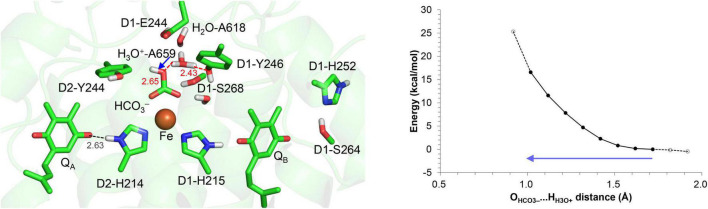
Potential energy profile for proton transfer from H_3_O^+^ at D1-Ser268 to the bicarbonate ligand. The blue arrow indicates the orientation of the proton transfer. Note that the geometry of each intermediate conformation is fully QM/MM optimized.

Although D1-Ser268 does not form an H-bond with the bicarbonate ligand (4.57 Å, Umena et al., 2011), the existence of a water molecule, which is only 2.44 Å away from the D1-Ser268 side chain (2.44 Å, Umena et al., 2011), is remarkable. The short distance might be due to the water molecule being H_3_O^+^ [e.g., O…O = 2.4 Å for typical H_2_O…H_3_O^+^ (Mikenda, 1986; Limbach et al., 2009; Figure 1A)]. However, assuming that the water molecule is H_3_O^+^ in the QM/MM calculation, H_3_O^+^ forms H-bonds with D1-Tyr246 and a water molecule adjacent to D1-Glu244, but not with D1-Ser268; this increases the O_D1–Ser268_…O_H3O+_ distance to 2.98 Å (Figure 5). Furthermore, H_3_O^+^ is unstable at this site, releasing the proton to ionized D1-Glu244. H_3_O^+^ can exist at the D1-Ser268 moiety only when D1-Glu244 is protonated. However, proton transfer from H_3_O^+^ at the D1-Ser268 to the bicarbonate ligand is energetically uphill (Figure 5), which suggests that D1-Ser268 is unlikely the proton donor to the bicarbonate. D1-Ser268 and the adjacent water molecule may be more likely to be involved in the proton transfer associated with the Q_B_H_2_ release, as suggested for mutant PSII proteins (Forsman and Eaton-Rye, 2020).

D1-Tyr246 does not form an H-bond with the bicarbonate –OH group (3.80 Å) in the PSII crystal structure (Umena et al., 2011), whereas D1-Tyr246 forms an H-bond with the bicarbonate –OH group (≈2.7 Å) in response to the bidentate-to-monodentate reorientation according to QM/MM calculations (Figure 3). D1-Tyr246 is located at the unique position, the interface between the channel that proceeds from the bicarbonate binding site (Figure 4) and the inner cavity, the Q_B_ pocket.

The elongation electron density near the hydroxyl group of D1-Tyr246 (Saito et al., 2013) may be interpreted as a peroxide O with the O–O distance of 1.48 Å, which can be fitted to the density (Figure 1A; Saito et al., 2013). Although the peroxide O is oriented toward the Q_B_ binding pocket, the link between the bicarbonate binding site and the Q_B_ binding pocket *via* D1-Tyr246 is weak due to the less polar O site.

**FIGURE 6:**
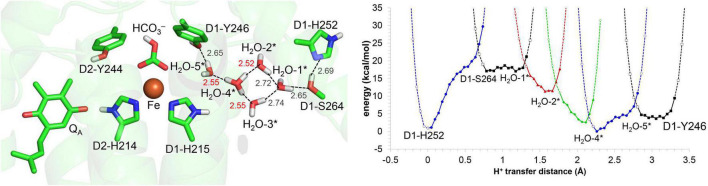
Potential energy profile for proton transfer in the Q_B_ binding pocket, adding water molecules (*: H_2_O-1 to 5). Note that the geometry of each intermediate conformation is fully QM/MM optimized.

Alternatively, the elongation of the density may be interpreted as H_3_O^+^. In the crystal structure reported by Uto et al. (2017), the O_D1–Tyr246_…O_water_ distance of 2.3 Å is closer to the O…O distance for a typical Zundel (H_2_O…H^+^…H_2_O) cation (≈2.3 Å), which suggests that the density may originate from H_3_O^+^ (Figure 1B). In this case, the H^+^ needs to enter *via* the Q_B_-exchange channel, possibly during the Q_B_ exchange. To investigate whether the uptake and existence of the H^+^ are energetically possible in the Q_B_ binding pocket, QM/MM calculations were performed, removing Q_B_ and filling the pocket with water molecules. The potential-energy profile indicates that the proton is energetically stable and populated among the two water molecules that bridge between D1-His215 and D1-Tyr246 (i.e., H_2_O-4* and H_2_O-5*), as H_3_O^+^ is stabilized by three H-bond acceptor groups (Figure 6). After Q_B_ re-enters the binding pocket, the H^+^ is allowed to exist only at the non-excluded water molecules, e.g., the one adjacent to D1-Tyr246. Indeed, the bulky methoxy groups of ubiquinone in PbRC are replaced with the methyl groups of plastoquinone in PSII, allowing one water molecules to remain at the D1-Tyr246 moiety. Thus, a water molecule can remain at the D1-Tyr246 moiety, the only polar site in the bottleneck of the hydrophobic Q_B_ pocket. This is likely the cause of the elongation of the electron density adjacent to the –OH region of D1-Tyr246.

**FIGURE 7:**
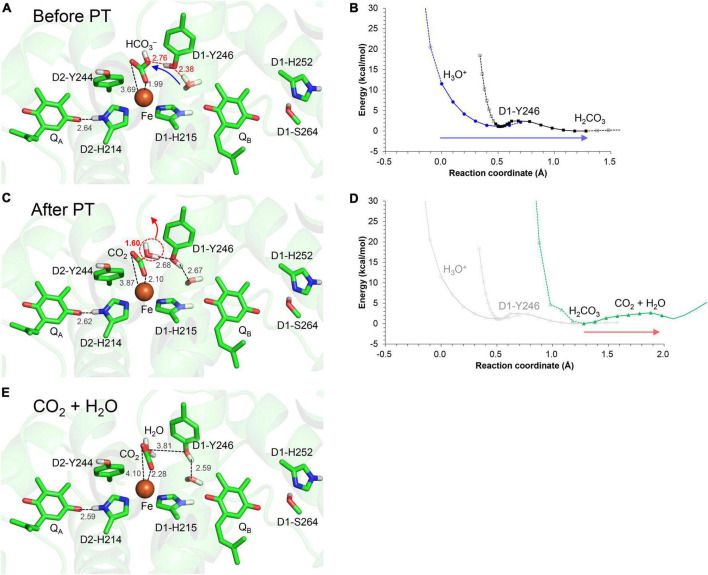
H-bond network *via* D1-Tyr246 to the monodentate bicarbonate ligand. **(A)** QM/MM-optimized geometry before proton transfer. **(B)** Potential energy profile for proton transfer along the H_3_O^+^…D1-Tyr246…bicarbonate H-bond network. **(C)** QM/MM-optimized geometry after proton transfer. **(D)** Potential energy profile for CO_2_ release from the monodentate binding site. **(E)** QM/MM-optimized geometry after decomposition to H_2_O and CO_2_. Blue arrows indicate the orientations of the proton transfer. Red arrows indicate the orientations of the structural changes. Note that the geometry of each intermediate conformation is fully QM/MM optimized.

When H_3_O^+^ is adjacent to D1-Tyr246, the bidentate-to-monodentate reorientation leads to the formation of an H-bond network that proceeds from H_3_O^+^ *via* D1-Tyr246 toward the monodentate bicarbonate (Figure 7A). The potential energy profile for the H-bonds suggests that the H-bond network can serve as a proton-transfer pathway (Figure 7B).

Intriguingly, when the proton arrives at the bicarbonate moiety along the Grotthuss-like proton conduit, the bicarbonate undergoes protonation (Figure 7C) followed by conversion to H_2_O and CO_2_ (Figure 7D). As the products move away from the Fe^2+^ site, the shape of O…C…O becomes linear, i.e., O = C = O (Figure 7E). Thus, the bicarbonate loss can occur through the conversion of the protonated bicarbonate to CO_2_ and H_2_O at the Fe^2+^ moiety (Figure 7D), which is in line with the CO_2_ release observed on the electron-acceptor side (Shevela et al., 2020).

The bidentate-to-monodentate reorientation of the bicarbonate ligand is a low-barrier process (Figure 3). In the monodentate bicarbonate ligand, the H_3_O^+^…D1-Tyr246…HCO_3_^–^/Q_A_^⋅–^ H-bond network is formed. The hydroxyl group forms a Grotthuss-like proton transfer pathway, accepting the proton from H_3_O^+^ and donating it to the bicarbonate. That is, D1-Tyr246 remains protonated during proton transfer, as suggested using Fourier transform infrared (FTIR) spectroscopy (Takahashi et al., 2009). The absence of the proton donor (H_3_O^+^), proton-conducting side chain (D1-Tyr246), and decomposable carboxylic ligand (bicarbonate) in PbRC implies that the proton-mediated Q_A_^⋅–^ stabilization occurs specifically in O_2_-evolving PSII for photoprotection. The involvement of D1-Tyr246 in the photoprotective role (proton-mediated Q_A_^⋅–^ stabilization) is in line with the findings of mutational studies; the photoautotrophic growth was slower in the D1-Y246F mutant PSII than in wild-type PSII (Kless et al., 1994); the D1-Y246A mutant PSII was also unable to grow photoautotrophically under strong light (Forsman et al., 2019).

The polar –OH region of D1-Tyr246 on the surface of the Q_B_ binding pocket can mediate the conduction of the proton to the bicarbonate binding site. The low-barrier bidentate-to-monodentate reorientation contributes to the formation of the “proton wire” (Stuchebrukhov, 2009) between D1-Tyr246 and the unligated –OH site of the bicarbonate. The corresponding H-bond formation with D1-Tyr246 does not occur upon the replacement of bicarbonate with formate, because formate has no –OH site. The absence of the –OH site may also be an origin of why formate substitution inhibits electron transfer from Q_A_ to Q_B_ (Eaton-Rye and Govindjee, 1988) or the exchange of Q_B_H_2_ (Sedoud et al., 2011). Thus, the hydrophobic and pre-organized protein electrostatic environment (Warshel, 1998; Warshel et al., 2006) on the bicarbonate binding site is likely to reduce the energy barrier for the bicarbonate protonation with respect to the bulk region, facilitating the direct protonation of the –OH site of the bicarbonate and the release of the product, gaseous CO_2_, without the formation of the typical (HO)_2_C = O intermediate (Loerting et al., 2000).

Molecular dynamics simulations suggested that the polar –OH site of the bicarbonate is linked to water molecules in the Q_B_ binding pocket *via* D1-Tyr246 during Q_B_H_2_ release (Sugo et al., 2022). These water molecules serve as a proton transfer pathway for the reprotonation of D1-His215 (Sugo et al., 2022), the proton donor during Q_B_H^–^/Q_B_H_2_ conversion (Saito et al., 2013, 2020). Thus, the link between the polar –OH site and D1-Tyr246 may provide a key to understand how the bicarbonate is associated with Q_B_H_2_ release, interacting with water molecules in the reprotonation pathway for D1-His215.

### Hydrophobic residues that form a CO_2_-releasing channel

QM/MM calculations suggest that the product, CO_2_, enters the channel that proceeds *via* D1-Glu244 and D1-Tyr246 toward the stromal protein surface (Figure 4). The entrance of the channel is hydrophobic, being formed by the side chains of D1-Tyr246, D2-Phe235, and D1-Ile248.

D1-Tyr246 may play two roles in the protonation of bicarbonate. While the polar –OH region mediates the conduction of the proton to the bicarbonate protonation site, the hydrophobic ring region can isolate the bicarbonate from the water molecules in the Q_B_ binding pocket, making the bicarbonate binding moiety hydrophobic. The loss of the solvation also increases p*K*_a_(HCO_3_^–^/H_2_CO_3_) with respect to the bulk water region, facilitating the conversion of bicarbonate to gaseous CO_2_.

## Conclusion

**FIGURE 8:**
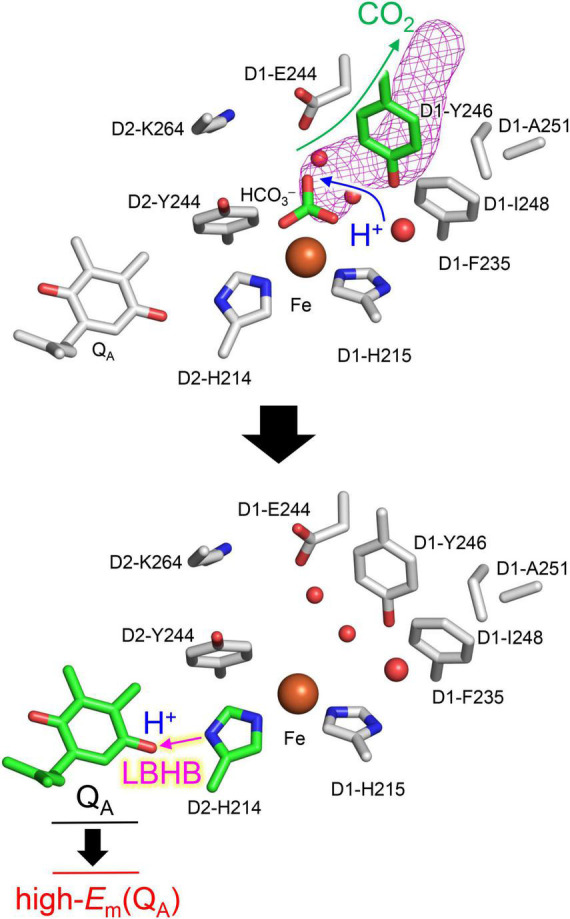
Overview of the proton-mediated photoprotection mechanism in photosystem II.

In response to the loss of the bicarbonate ligand, a short low-barrier H-bond forms between D2-His214 and Q_A_^⋅–^, which facilitates the proton migration toward Q_A_^⋅–^ and increases *E*_m_(Q_A_) (Figure 2). The loss of bicarbonate may be induced by the protonation. The D1-Glu244 conformation identified in the PSII crystal structure does not support the role in donating a proton to the bicarbonate ligand, whereas D1-Tyr246 may facilitate bicarbonate protonation and decomposition into H_2_O and CO_2_ (Figure 7). The channel, which is formed by D1-Tyr246, D2-Phe235, and D1-Ile248, may serve as a CO_2_-releasing channel (Figure 4). These constitute the basis for the photoprotection mechanism through the high-*E*_m_(Q_A_) conformation, which inhibits the backward electron transfer from Q_A_^⋅–^ *via* the triplet-generating pheophytin/chlorophyll cofactors (Figure 7). The proton-mediated photoprotection mechanism is pronounced exclusively in PSII, as the bicarbonate ligand is replaced with Glu-M234 in PbRC from *Rhodobacter sphaeroides* (Figure 8). The mechanism of the low-barrier H-bond formation caused by the loss of the bicarbonate ligand may also be associated with the Q_A_H_2_ formation under high light in PSII (Vass et al., 1992; Noguchi, 2002), as Glu-M234 exists permanently and Q_A_ never leaves in PbRC (Wei et al., 2022). These findings could elucidate how the type-II reaction centers show notable structural differences not only on the lumenal oxygen-evolving side but also on the stromal electron acceptor side.

## Data availability statement

The original contributions presented in this study are included in the article/Supplementary material, further inquiries can be directed to the corresponding author.

## Author contributions

HI designed the research and wrote the manuscript. YS and HI performed the research and analyzed the data. Both authors contributed to the article and approved the submitted version.
